# Social capital, education, and subjective well-being in Ecuador

**DOI:** 10.3389/fsoc.2024.1417538

**Published:** 2024-08-21

**Authors:** Aracelly Núñez-Naranjo, Ximena Morales-Urrutia, Luis Simbaña-Taipe

**Affiliations:** ^1^Centro de Investigaciones de Ciencias Humanas y de la Educación (CICHE), Universidad Indoamérica, Ambato, Ecuador; ^2^Facultad de Contabilidad y Auditoría, Universidad Técnica de Ambato, Ambato, Ecuador; ^3^Departamento de Ciencias Administrativas, Económicas y de comercio, Universidad de las Fuerzas Armadas, Sangolquí, Ecuador

**Keywords:** affiliation sindical, economics, political science, social sciences, union membership

## Abstract

The study examines the interaction between social capital, education, and subjective well-being in Ecuador, highlighting its impact on economic development. The study aims to understand the situation of social capital and subjective well-being and how the identified factors explain the impact on subjective well-being in the Ecuadorian population, using a descriptive and analytical approach with information from the World Value Survey database of waves 6 and 7. The main results show a significant relationship between social capital and subjective well-being, with positive influences such as justice and union membership, and negative effects of public administration and media. In conclusion, the importance of strengthening social capital and improving public services and communication to promote the well-being of the Ecuadorian population is emphasized.

## 1 Introduction

In recent decades, the concepts of subjective well-being and social capital have garnered special attention from the social sciences, economics, and political sciences, due to their significant contributions to the development and economic growth of nations (Clark et al., 2019). In this regard, Calcagnini and Perugini (2019) mention that social capital directly influences this phenomenon by contributing to aspects related to trust, networks, and norms (Coleman, 1988; Putnam, 1993; Bourdieu, 2006), which, in turn, facilitate cooperation and coordination for mutual benefit. Therefore, the authors consider social capital as a set of people who establish horizontal relationships; that is, what is known as networks or associated norms that enhance the productivity of a community.

Similarly, the results shown in previous research have identified a series of factors related to subjective well-being and social capital, among which are highlighted, for example, family situation, economic, and social resources (Hommerich and Tiefenbach, 2018). In this regard, social resources are considered as the main drivers of subjective well-being (Helliwell and Putnam, 2004), which in turn presents different dimensions that are directly related to the concept of social capital.

In recent decades, social capital has undergone significant changes, becoming a multidimensional construct (van Oorschot and Arts, 2005). Coleman (1988) considers that social capital acts as a facilitator in the relationships of different actors, be they individuals or companies. Similarly, Putnam (1993) emphasizes that social capital has more of a collective character; that is, it describes the characteristics of organizations in the social aspect; such as networks, norms, and trust.

### 1.1 Definitions and main generalities of social capital

The concept of social capital originated in the 19th century based on ideas proposed by Durkheim and Adam Smith (Lin, 2017). This concept paid special attention to the characteristics of public capital belonging to a specific country or society that possesses public goods (Helliwell and Putnam, 2004). However, it wasn’t until the 1950s that social capital was considered as a subject that sparked sociological and pedagogical research.

Although there is a considerable amount of contributions regarding the definition of social capital, the concepts that have gained the most recognition focus on three fundamental aspects: trust, norms, and networks (Bourdieu, 2012; Coleman, 1988; Putnam, 1993). In this regard, Bourdieu (1980) mentioned that social capital represents social networks and their connections, highlighting mutual knowledge and recognition. On the other hand, Coleman (1990) defines it as a facilitator of actions by actors within a social structure; in other words, social exchange. Meanwhile, Putnam (1993) emphasizes the collective nature of social capital, in the sense that it describes the social characteristics and interactions among individuals or social groups that facilitate cooperation and coordination for mutual benefit (Hommerich and Tiefenbach, 2018; Calcagnini and Perugini, 2019).

On the other hand, Fiorillo and Nappo (2014) mention that social capital is defined by its function; it is not a single entity but a variety of them, which share two common characteristics: firstly, they all consist of some aspect of social structure; and secondly, they facilitate certain actions of individuals who belong to a structure.

Gilbert et al. (2014), argue that the concept of social capital is composed of two components: one structural and the other cognitive. Regarding the former, it refers to the actual social network; that is, the size and intensity of contacts (family, friends, and coworkers); and, on the other hand, to the subjective perception of one’s own social resources (perceived social support, trust in institutions, and social trust).

### 1.2 Dimensions of social capital

#### 1.2.1 Structural dimension of social capital

The structural dimension entails the configuration and pattern of connections among individuals, represented by the existence of social networks (who knows whom), roles, institutions, relationships, and procedures within a group (Claridge, 2018).

The structural dimension of social capital points to the benefits derived from information and assistance obtained from social networks or groups of individuals. Additionally, aspects such as density, connectivity, hierarchy, and appropriateness within a specific context—be it a group, an organization, or a community—are considered from these networks (Davenport and Daellenbach, 2011). In this regard, Lefebvre et al. (2016) have analyzed that networks also exhibit strength, centrality of ties, stability, and size.

Furthermore, according to Tsai and Ghoshal (1998), the structural dimension serves as a precursor to the cognitive and relational dimensions, as the authors explain that social relationships and structures must be established prior to social exchange. Therefore, the connections forged within networks facilitate social interaction, which in turn promotes the development of the intangible dimensions of social capital (Gooderham, 2007).

#### 1.2.2 Cognitive dimension of social capital

In this dimension of social capital, resources are related to interpretations, representations, and shared systems of meaning among parties (Nahapiet and Ghoshal, 1998). Cognitive social capital represents the language and different codes shared in the communication process; that is, the meaning conveyed through common vocabularies and narratives (Davenport and Daellenbach, 2011). Additionally, Inkpen and Tsang (2005) and Tsai and Ghoshal (1998) describe it as the vision, objectives, and culture shared by a society. Therefore, this dimension is intangible, as it is the result of the interpretation of shared reality.

In this regard Bourdieu (2012), relates this dimension to his theory of habitus, stating that it is a set of acquired practices, reflexes, and behaviors that individuals put into practice in society. Similarly, Sitton (2003) relates it to Habermas’s theory of the lifeworld, which considers attitudes, practices, and competencies in terms of each person’s cognitive horizon.

Cognitive social capital is manifested through the use of specific language and codes that have meaning within an organization; however, outside of it, they lose their relevance or sense (Ansari et al., 2012).

#### 1.2.3 Relational dimension of social capital

This dimension refers to the characteristics and qualities of personal relationships, emphasizing trust, norms, obligations, identification, among others (Nahapiet and Ghoshal, 1998).

Lefebvre et al. (2016), mention that the relational dimension is related to the nature and quality manifested in relationships, which, in turn, are the product of interactions and are expressed through attributes related to behavior, such as group norms, obligations, and identification.

Similarly, the relational dimension is directly related to trust, reliability (Putnam, 1993), shared norms and sanctions (Coleman, 1990), obligations and expectations (Coleman, 1990; Burt, 1999), and identity and identification (Merton and Merton, 1968) with other individuals within society.

One fundamental aspect of the relational dimension is that it fosters associability, meaning the willingness to prioritize collective goals over individual ones (Claridge, 2018).

Overall, from the perspective of scholars in the field, both the social and relational dimensions are intangible and are related to perception and opinion, making them somewhat subjective and variable among different individuals and contexts. In this regard, the main difference highlighted is that the relational dimension is embedded in social relationships, while the cognitive dimension describes the characteristics of social context in a broader sense. Therefore, Claridge (2018) mentions that shared understanding within a community falls into the cognitive realm, whereas trust and norms of reciprocity correspond to the relational dimension, as they describe the quality of relationships developed at the social level.

### 1.3 Subjective well-being

Currently, well-being is considered one of the main objectives within a state’s public policies, as it encompasses an individual’s state of comfort, behavior, and emotional expression (Omodei and Wearing, 1990). Nordbakke and Schwanen (2014) mention that well-being presents three types of dimensions.

Firstly, a differentiation is made between subjective and objective well-being. Subjective well-being evaluates a person’s quality of life based on perception and experience. Meanwhile, objective well-being is based on the assessment of the environment in which people develop, according to different criteria, norms, values, goals, and purposes (Hommerich and Tiefenbach, 2018).

Secondly, from applied philosophy, two approaches related to well-being are considered: hedonism and eudaimonism. Hedonism maintains a more utilitarian view, considering that a person’s well-being comes from the satisfaction, dissatisfaction, or pain they experience (Diener et al., 2017). In contrast, eudaimonia emphasizes activities related to goals, the meaning of life, and self-realization (Ryan and Deci, 2011).

Thirdly, stability is considered. In this regard, well-being can be classified into universalism and contextualism. Universalism promotes a form of thought aimed at the stability of well-being, regardless of the standards applied in different countries. On the other hand, contextualists maintain that the levels and components by which well-being is formed could vary according to desires and cultural environment (Hommerich and Tiefenbach, 2018).

In this context, some authors have related the concept of subjective well-being to variables such as life satisfaction, happiness, economic stability (income), social, psychological (personality), cultural (beliefs and relationships), natural environment, among others (Helliwell et al., 2017; Dai, 2018).

### 1.4 Subjective well-being and happiness

According to the OECD (2015), the term “happiness” is closely related to emotional well-being, which, in turn, is expressed through positive affect (emotion, joy) and negative affect (sadness, pain, anger, etc.). From this perspective, there is a perception that a person is doing well when they experience more positive feelings than negative ones (Joo and Lee, 2017).

In this regard, Weimann et al. (2015) associate happiness with factors such as wealth (income), health, job stability, positive relationships, and personal happy events, which are in turn related to higher subjective well-being. Regarding the latter, Diener (1984) states that subjective well-being is colloquially referred to as happiness and refers to how each person evaluates their life; in other words, individuals experience higher subjective well-being when they feel pleasant emotions, engage in activities of interest, and feel satisfaction with the life they lead (Clark et al., 2019).

In the same line, authors like Andrews and McKennell (1980) and Diener (1984) describe happiness through two processes. The first, upward, implies that the existence of happiness depends on positive and negative feelings, and the second, downward, in which happiness is the result of different subjective evaluations of human life experiences (Diener and Oishi, 2005).

On the other hand, Acosta-González and Marcenaro-Gutiérrez (2021) mention that happiness results from two components: (a) short-term, depending on recent events in a person’s life; and (b) long-term, which will depend directly on entertainment or health activities.

Finally, Veenhoven (2012) describes happiness as the degree of satisfaction with life (income, marital status, personality, beliefs, attitudes, relationships, economic, social, and natural environment, among others) that each person has, considering happiness and satisfaction to be synonymous concepts (Hommerich and Tiefenbach, 2018; Medvedev and Landhuis, 2018).

## 2 Methodology

The approach applied was, on one hand, descriptive, as it aimed to combine criteria to understand the situation of social capital and subjective well-being in the selected periods for analysis. On the other hand, it was analytical, as it aimed to advance the study of social capital and subjective well-being and their explanatory factors through statistical analysis techniques.

The completion of this work also relied on documentary-bibliographic research and the use of secondary statistical sources, which were systematically treated to develop the state of the art.

This study utilized individual-level information (collected and standardized data) from the World Values Survey Association (2020), years that allow understanding the situation of the studied variables but not establishing a comparative relationship between years, variables, or territories, as the interest is to examine the reality of the Ecuadorian case. It should also be mentioned that this organization regularly conducts a survey validated by national and international experts in different countries worldwide, aiming to measure the experiences of 129,000 citizens in 77 countries participating in this survey, including approximately 1,200 individuals from Ecuador. In this regard, the selected database constitutes a robust data source due to the trajectory and recognition of the institution collecting the data and the experts validating the questionnaires; therefore, it is considered an optimal empirical basis for addressing this study.

The mentioned database includes 300 indicators from a standardized questionnaire structured into 14 themes related to Happiness and well-being, Social capital, trust, and organizational membership, Perceptions about science and technology, Social values, norms, stereotypes, among others. From the aforementioned, indicators related to the theoretical review and specifically the case of Ecuador were selected.

**Figure 1:**
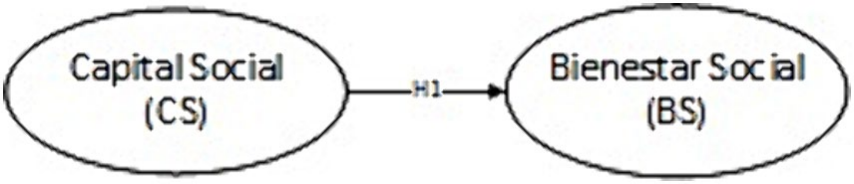
Conceptual model: graphical description.

Figure 1 represents the conceptual model, proposing, as research hypothesis (H1), the relationship between the latent variable Social Capital (SC) and Social Well-being (SW).

For data processing, both Wave 6 and Wave 7 databases were downloaded, and the data were cleaned and filtered for the analyzed variables and the country under study. The scales used in the data collection instrument maintained different measurement scales, and for this particular case, they were standardized to a 4-level scale. With the cleaned database, it was entered into the selected statistical software to perform various tests that confirmed the suitability of applying Structural Equation Modeling to explain the analyzed phenomenon.

Some limitations of this study should be mentioned, particularly the collection of primary data sources due to spatial, temporal, and economic constraints. However, the data presented here constitute a preliminary approach to analyzing these phenomena in the specific case of Ecuador.

## 3 Results

In order to measure the reliability of the measured scale, the Cronbach’s Alpha coefficient was evaluated using SPSS 25 software (Table 1). The measurement accuracy for the variable SC (0.833) is acceptable. On the other hand, regarding the variable SW (−0.210), it yields a negative result, which is due to a negative average covariance among items (observed variables), contradicting reliability assumptions.

**Table 1:** Confirmatory factor analysis: summary of the measurement model, validity, and reliability.

	Standardized loadings
**Social Capital (SC) (CA = 0.833)**	
Your family	0.160
Your neighborhood	0.226
People you know personally	0.255
People you meet for the first time	0.154
People of another religion	0.202
People of another nationality	0.219
The armed forces	0.535
The press	0.602
Television	0.564
Labor unions	0.625
The police	0.601
The courts	0.688
The government	0.569
Political parties	0.623
The civil service	0.612
Charitable or humanitarian organizations	0.357
The united nations	0.440
**Subjective Being (SB) (CA = −0.203)**	
Taking all things together	−0.540
All in all	−0.500
All things considered	0.495
How satisfied are you with the financial	0.469
**Model fit (summary)**	
Chi-square = 579.086, df = 175, *p* = 0.000, *χ*^2^/gl = 3.3 CFI = 0.939; IFI = 0.939; TLI = 0.927; NFI = 0.915 RMSEA = 0.044	

CA, Cronbach Alfa.

Based on the complexity of the model and the need to test the relationship between the established constructs (SC and SW), the structural equation modeling (SEM) approach using the maximum likelihood method was employed. The estimation of the hypothetical model is shown in Table 2.

**Table 2:** Structural equation model: summary of model fit and estimated parameters.

Hypothesis	Causal relationships	Standardized estimated parameters		Test
H1	CS à BS	−0.089	*	Supported
	Model fit (summary)			
	Chi-square = 579.086, df = 175, *p* = 0.000, *χ*^2^/gl = 3.3 GFI = 0.954, RMSEA = 0.044 CFI = 0.939; IFI = 0.939; TLI = 0.927; NFI = 0.915; AGFI = 0.939			

*p < 0.05; **p < 0.01; ***p < 0.001; ns, no significativo.

SC, Capital Social; SB, Bienestar Subjetivo.

The absolute fit measures indicate that the model adequately predicts the observed covariance or correlation matrix. Thus, the obtained value of the Chi-square to degrees of freedom ratio (*χ*^2^/df = 3.3), the Goodness of Fit Index (GFI = 0.954), demonstrate an acceptable fit, and the Root Mean Square Error of Approximation (RMSEA = 0.044) indicates that the results in the sample can be inferred to the studied population. Regarding the incremental fit measures, Comparative Fit Index (CFI = 0.939) shows that the non-centrality measures are acceptable; Incremental Fit Index (IFI = 0.939), Tucker-Lewis Index (TLI = 0.927), Normed Fit Index (NFI = 0.915), and Adjusted Goodness of Fit Index (AGFI = 0.939) demonstrate acceptable values.

**Figure 2:**
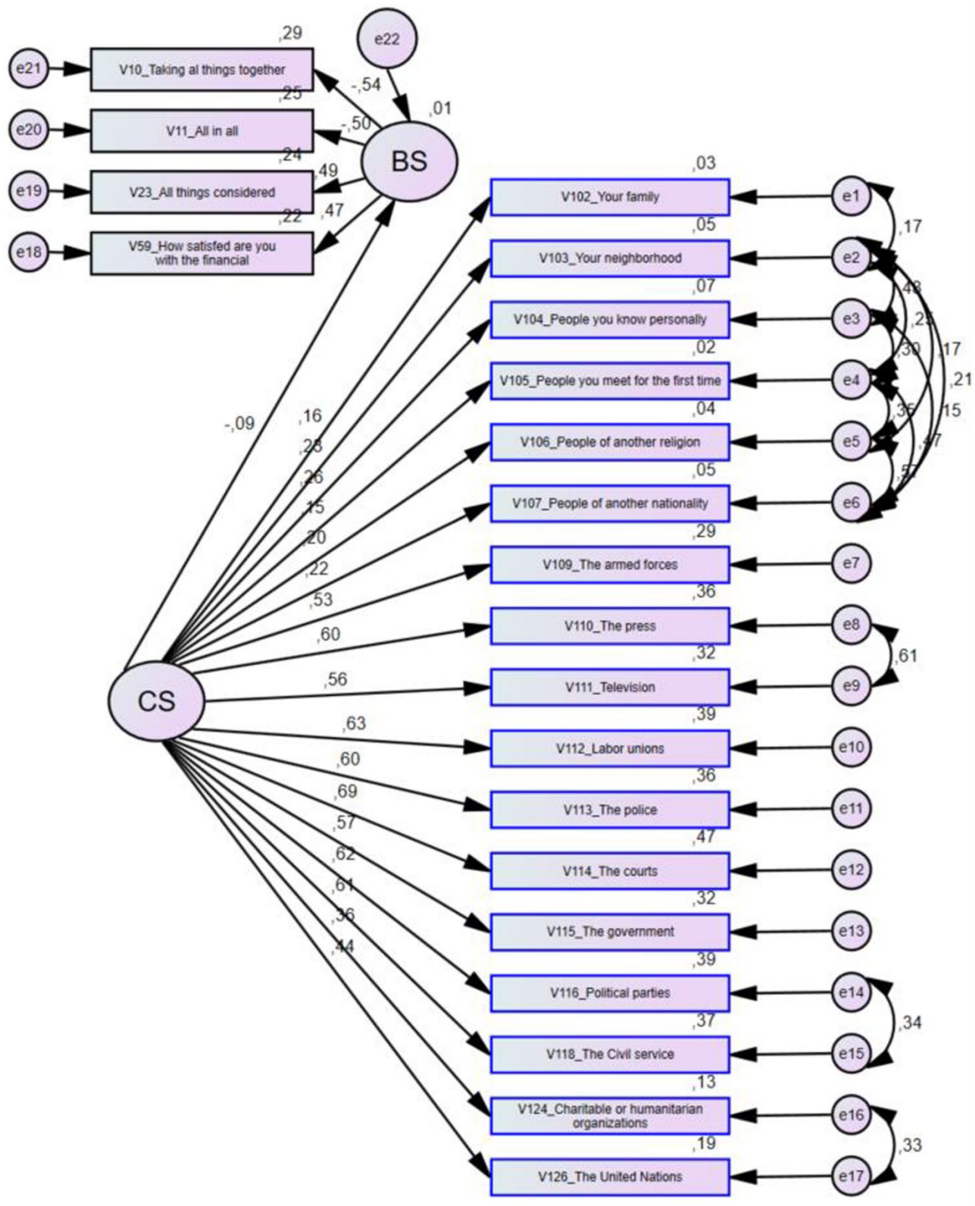
Conceptual model: graphical description with estimated parameters.

The results obtained from the standardized structural model are shown in Figure 2, which graphically represents the model’s relationships and estimated parameters with an acceptable level of significance.

As observed in Figure 2, social capital has a negative impact on social well-being, from which it can be established that the factor with the greatest relevance within social capital is justice with a score of 0.69, followed by labor associations with 0.63, political parties with 0.62, public administration with 0.61, and the press and police with 0.60. Other significant factors include government with 0.57, television with 0.56, and the armed forces with 0.53.

## 4 Discussion

### 4.1 Dimensions of social capital in relation to subjective well-being

The relevance of the impact of justice on social well-being aligns with what Rodriguez-Serpa et al. (2023) state, who assert that the legitimate trust placed in the judicial decisions of Constitutional Courts, which have ethical and moral implications, generates a positive impact on people’s well-being. Similarly, Wenner and Campbell (2017) determine that the existence of norms, valid for everyone and respected by individuals, allows for achieving social well-being by minimizing uncertainty and making life predictable. The aforementioned results are consistent with what Sundaresh et al. (2020) establish, who suggest that exposing citizens to the justice system is associated with a decrease in social well-being, necessitating the need for financial stability and social support to help individuals recover their well-being after involvement with the justice system. However, Botero (2020) suggests that these results are general factors that can vary the exact relationship, as it may change when other factors intervene, such as economic well-being, equality, and quality of life.

Regarding the variable of labor associations, Blanchflower and Bryson (2020) establish that union membership has a positive impact on the well-being of employees, considering that union members are less likely to feel stressed, worried, depressed, or lonely. These results align with those established by Qi et al. (2021), who state that union practices can effectively help reduce adverse effects of high-performance work systems, ensuring the well-being of employees in the workplace. This implies that individuals affiliated with a labor association experience higher job satisfaction (Blanchflower and Bryson, 2020), which contributes to improving their subjective well-being. There are various dimensions related to work, such as well-being, trust, time, and macroeconomic issues. However, employees who are affiliated with a union are more satisfied. These findings confirm that union status has a positive relationship with job satisfaction and overall well-being of individuals.

Regarding the variable of political parties, it is important to highlight that subjective well-being as a political objective provides evidence of the national happiness levels generated by the electoral fate of incumbent governments in general elections, as well as whether individual happiness can explain voting intentions, as established by Ward (2020).

Toshkov and Mazepus (2023) analyze the effects of winning or losing democratic elections and establish that being on the losing side is related to being less happy. Similarly, Pinto et al. (2021) argue that those who identify with the losing party experienced a strong negative effect on subjective well-being, whereas little or no impact was observed on well-being among those who identified with the winning team. Additionally, Ward (2020) states that subjective well-being directly impacts electoral outcomes, as it explains a greater variance in the percentage of votes for governing parties.

Regarding the variable of public administration, Mu et al. (2023) determine a positive effect of basic services and public services on subjective well-being. Conversely, Ding et al. (2022) identifies a negative effect of public services on happiness, suggesting that changes in public services can produce significant variations in citizens’ perceptions. Meanwhile, Moncayo Vives (2019), analyzing the impact of public investment on well-being, establishes that this variable stimulates the economy and consequently improves the quality of life of the population.

Regarding the press variable, according to El-Ibiary and Calfano (2022), it can have a positive impact depending on the approach used, especially when conceiving happiness as a tool to face life’s challenges. This perspective also aligns with the findings of Aedo et al. (2020).

Regarding the police variable, Al Kaabi (2022) establishes that perceptions and characteristics of police work are determinants of happiness. These results are consistent with those found by Wijaya et al. (2021). Meanwhile, Petchtam and Limwongsakul (2022) argue that police presence in the community has a positive effect as it reduces fear of crime and increases trust and peace of mind in citizens’ lives.

As for the government variable, Momeni Mahmouei and Razmi (2023) argue that government size below the threshold of human development leads to a decrease in societal happiness; however, crossing this threshold increases societal happiness. Helliwell et al. (2020), establish that the quality of government provision is correlated with national happiness, a finding consistent with that of Liu et al. (2022).

Finally, television contributes to a positive impact, as explained by Lüders (2022), who establishes that streaming and agency are associated with enjoying television due to its social importance and immersive viewing, leading to a positive impact. These findings are related to those of Chadi and Hoffmann (2021), who establish that television is a factor that increases happiness. Therefore, television is an economical and effective option as a political instrument. Contrary to these results, Guillen-Royo (2019) argues that dedicating a high consumption to television leads to lower life satisfaction.

## 5 Conclusion

In Ecuador, participation in community organizations, trust in institutions, and strong social networks can progressively contribute to the increase in subjective well-being of citizens. Additionally, the quality and impartiality of the media play a crucial role in shaping public opinion.

Regarding justice and its relationship with subjective well-being, it indicates that it does not foster trust and suggests that the rules are not clear, and moreover, judicial decisions lack legitimacy. Likewise, belonging to an association or union that lacks adequate support or assistance shows a negative impact on the well-being of workers. On the other hand, the non-compliance by political parties generates a negative impact on subjective well-being, derived from the continuous violation of campaign promises, as well as the corruption observed within political groups.

Regarding public administration and government, they show a negative effect on subjective well-being, probably due to the prevalent mismanagement in most public institutions and the mishandling of resources, resulting in limited and low-quality public services for citizens. Conversely, the security provided by the police has a positive effect on subjective well-being, as they contribute to creating a safer environment, which may influence better economic and recreational activities within the country. Finally, the media, specifically television, has a negative impact on well-being because the content displayed on screens is often violent or unpleasant, leading to a feeling of dissatisfaction and displeasure with the content consumed through television media.

Subjective well-being in Ecuador, analyzed through a series of factors, does not present a favorable outlook for individuals. The combination of factors present in Ecuadorian society seemingly meets the conditions conducive to generating well-being and improving the overall quality of life of the population.

## Data availability statement

The original contributions presented in the study are included in the article/supplementary material, further inquiries can be directed to the corresponding author.

## Author contributions

AN-N: Conceptualization, Funding acquisition, Investigation, Project administration, Resources, Supervision, Validation, Writing – review & editing. XM-U: Conceptualization, Data curation, Formal analysis, Visualization, Writing – original draft. LS-T: Conceptualization, Formal analysis, Methodology, Visualization, Writing – original draft.
